# Roles of adjuvant and route of vaccination in antibody response and protection engendered by a synthetic matrix protein 2-based influenza A virus vaccine in the mouse

**DOI:** 10.1186/1743-422X-4-118

**Published:** 2007-10-31

**Authors:** Krystyna Mozdzanowska, Darya Zharikova, Mare Cudic, Laszlo Otvos, Walter Gerhard

**Affiliations:** 1 Immunology Program, The Wistar Institute, Philadelphia, USA; 2 Department of Pathology and Laboratory Medicine, University of Wisconsin Hospital and Clinics, Madison, USA; 3 Department of Chemistry and Biochemistry, Florida Atlantic University, Boca Raton, USA; 4 Temple University, Sbarro Institute, Philadelphia, USA

## Abstract

**Background:**

The M2 ectodomain (M2e) of influenza A virus (IAV) strains that have circulated in humans during the past 90 years shows remarkably little structural diversity. Since M2e-specific antibodies (Abs) are capable of restricting IAV replication in vivo but are present only at minimal concentration in human sera, efforts are being made to develop a M2e-specific vaccine. We are exploring a synthetic multiple antigenic peptide (MAP) vaccine and here report on the role of adjuvants (cholera toxin and immunostimulatory oligodeoxynucleotide) and route of immunization on Ab response and strength of protection.

**Results:**

Independent of adjuvants and immunization route, on average 87% of the M2e-MAP-induced Abs were specific for M2e peptide and a variable fraction of these M2e(pep)-specific Abs (average 15%) cross-reacted with presumably native M2e expressed by M2-transfected cells. The titer of these cross-reactive M2e(pep-nat)-specific Abs in sera of parenterally immunized mice displayed a sigmoidal relation to level of protection, with EC_50 _of ~20 μg Ab/ml serum, though experiments with passive M2e(pep-nat) Abs indicated that serum Abs did not fully account for protection in parenterally vaccinated mice, particularly in upper airways. Intranasal vaccination engendered stronger protection and a higher proportion of G2a Abs than parenteral vaccination, and the strength of protection failed to correlate with M2e(pep-nat)-specific serum Ab titers, suggesting a role of airway-associated immunity in protection of intranasally vaccinated mice. Intranasal administration of M2e-MAP without adjuvant engendered no response but coadministration with infectious IAV slightly enhanced the M2e(pep-nat) Ab response and protection compared to vaccination with IAV or adjuvanted M2e-MAP alone.

**Conclusion:**

M2e-MAP is an effective immunogen as ~15% of the total M2e-MAP-induced Ab response is of desired specificity. While M2e(pep-nat)-specific serum Abs have an important role in restricting virus replication in trachea and lung, M2e-specific T cells and/or locally produced Abs contribute to protection in upper airways. Intranasal vaccination is preferable to parenteral vaccination, presumably because of induction of local protective immunity by the former route. Intranasal coadministration of M2e-MAP with infectious IAV merits further investigation in view of its potential applicability to human vaccination with live attenuated IAV.

## Background

Two types of influenza A virus (IAV) vaccines are currently used: 1) non-infectious preparations of detergent-disrupted virus particles or purified viral glycoproteins, hemagglutinin (HA) and neuraminidase (NA), which are licensed for all ages ≥0.5 y and 2) live attenuated, temperature sensitive and cold-adapted IAV, which are currently licensed for vaccination of 5 to 49 y old subjects [1]. Both vaccines attempt to engender strong Ab responses to HA and NA, and can be 70–90% effective in preventing IAV-induced illness [1]. Still, current vaccines have shortcomings: First, the viral glycoproteins are highly variable targets and change from year to year. Thus, the efficacy of current vaccines depends greatly on how well the glycoproteins of the vaccine strains, which must be selected 8–9 months prior to the influenza season, match those of the actual circulating epidemic strain. A mismatch is likely to cause a decrease in protective efficacy. Second, the presently licensed inactivated vaccines have relatively low (≤50%), if any [2], protective efficacy in the elderly (≥60 y). This is a problem because elderly people are at high risk for severe disease, and 90% of influenza-associated mortality in the U.S. (on average ~30,000/year) occurs in this segment of the population [1]. Third, newborns (≤0.5 y), who also are at high risk for severe disease and are usually protected by passively acquired maternal Abs [3], may be with no or low protection in case of a major mismatch between vaccine and circulating IAV strains. These shortcomings of current vaccines could be lessened by a vaccine or vaccine adjunct that engendered protective Abs against viral structures of low or no variability, and thereby provided a constant level of long lasting resistance against IAV infection, independent of the glycoprotein makeup of circulating IAV strains.

The ectodomain of matrix protein 2 (M2e) is a promising candidate for a broadly protective IAV vaccine as M2e underwent remarkably little sequence variation amongst human IAV strains isolated between 1918 to 2005, and M2e-specific Abs have been shown to display significant protective activity in animal models [4-11]. Most importantly, however, M2e-specific Ab titers are very low or undetectable in human sera, suggesting that current vaccines or recovery from natural infection fail to induce significant M2e-specific Ab responses [12-14]. Thus, humans are currently without significant M2e-specific Ab-mediated protection. Based on these premises, various M2e-specific vaccine constructs have been explored in recent years and tested for immunogenicity and protective activity in preclinical models [4-6,8,9,15-18]. In view of the relatively small size of M2e (23aa), we chose to develop a synthetic multiple antigenic peptide (MAP) vaccine. The latter consists of four M2e and two helper T cell peptides linked to a linear scaffold peptide [17]. In a previous study, we showed that immunization of mice with M2e-MAP plus cholera toxin (CT) and immunostimulatory oligodeoxynucleotide (ODN) by the i.n. route induced significant M2e-specific Ab responses and protection [17]. Here, we report studies in which we investigated the roles of adjuvant and route of vaccine administration on titer and composition of the M2e-specific Ab response and strength of protection.

## Results

### Specificity of the M2e-MAP-induced Ab response

**Figure 1:**
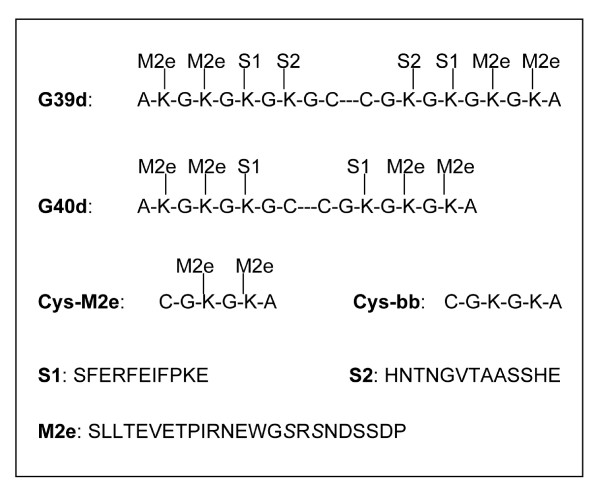
**Composition of MAPs**. The amino acid (aa) composition of the scaffolds of G39d and G40d is shown in single letter code. The triple dash in the scaffolds denotes the disulfide bond between adjacent cysteins. S1 and S2 are helper T cell peptides and M2e the 24 N-terminal aa of M2, linked through their C-terminal aa to the indicated lysines of the scaffold peptides.

**Figure 2:**
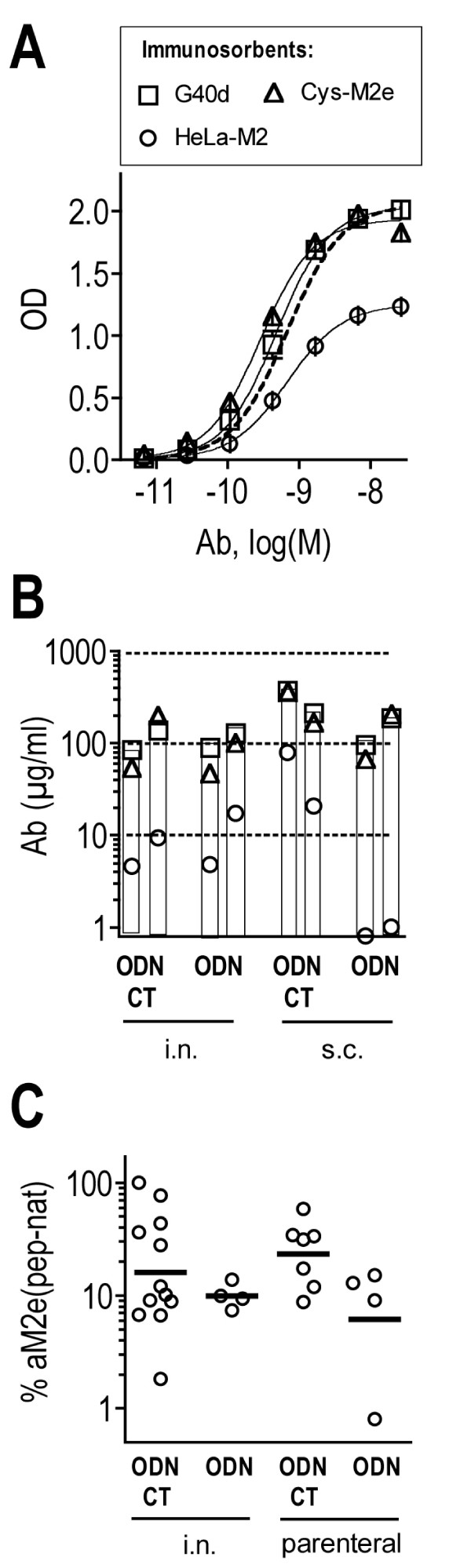
**Fine specificity of the M2e-MAP induced Ab response**. A. MAb 14C2-S1-4, which was used in all assays for quantification of serum Ab titers, was tested in ELISA against M2e-MAP Gd40 (squares), Cys-M2e (triangles) and HeLa-M2 (circles) as described in the method section, using the same reagents and incubation times for each assay. The mean OD (± SEM) above background of six replicates at each Ab dilution are shown. The three sigmoidal titration curves have similar EC50 values (-9.3 vs G40d, -9.5 vs Cys-M2e, -9.2 vs HeLa-M2). To further demonstrate the similarity between the three titration curves, OD values measured against HeLa-M2 were multiplied by 1.65 to generated the stipulated curve. A representative assay is shown. B. Pooled plasma samples (5 mice/group), obtained 3 wks after second (left column) and third (right column) immunization, were tested by ELISA for M2e-MAP- (squares), M2e(pep)- (triangles) and M2e(pep-nat)-specific (circles) Ab titers as described in the method section. The mice had been immunized with 3 μg M2e-MAP G40d and adjuvants by i.n. or s.c. routes as indicated below the x axis. Each symbol shows the mean serum Ab concentration determined in each sample by 2–3 independent assays. Data from a single vaccination experiment are shown. C. The fraction of M2e(pep-nat)-specific Abs is expressed as percent of the M2e(pep)-specific Ab concentration within each sample. Each dot indicates the % of anti-M2e(pep-nat) per group of 3–5 mice immunized by one of the protocols indicated below the x axis. In most groups, samples from secondary and tertiary responses were tested, and the mean % of these is shown. Horizontal bars indicate the geometric means within a vaccination protocol. Data from 12 independent vaccination experiments are shown. Groups immunized by different protocols did not differ significantly (ANOVA) with regards to percentage of anti-M2e(pep-nat)-specific Abs.

M2e-MAP consists of a scaffold peptide to which M2e- and Th determinant peptides are covalently attached (Fig 1). Each of these peptides or combinations thereof may serve as target for MAP-induced Abs. We were interested in learning what fraction of the total M2e-MAP-induced Ab response was specific for M2e peptide and what fraction of the M2e-peptide-specific Abs was capable of binding to native tetrameric M2e. The latter was of particular interest because only Abs capable of binding to native tetrameric M2e would be expected to display protective activity. To measure the total M2e-MAP-specific response, we tested sera of M2e-MAP-immunized mice by ELISA against wells coated with the M2e-MAP used for immunization as specific and uncoated (BSA-blocked) wells as non-specific (background) immunosorbents. M2e-peptide (pep)-specific Ab titers were measured by using Cys-M2e coated wells as specific and Cys-bb-coated wells as non-specific immunosorbents. Abs specific for cell-expressed, presumably native, tetrameric M2e were measured by using HeLa-M2 cells as specific and HeLa-C10 cells as non-specific immunosorbents. Since the latter Abs are a fraction of the M2e(pep)-specific Abs, they will be referred to as M2e(pep-nat)-specific to distinguish them from Abs that react with native cell-expressed M2e but not with M2e peptide, an Ab population detected in mice that have recovered from repetitive IAV infections [13]. The M2e-specific MAb 14C2-S1-4, which binds with comparable efficacy to all three specific immunosorbents under the present assay conditions (Fig 2A), was used as a standard to quantify the ELISA data.

Fig 2B shows results from an experiment in which four groups of mice were immunized three times by i.n. or s.c. routes with the M2e-MAP G40d together with the immunostimulatory oligodeoxynucleotide 1826 (ODN) or ODN and cholera toxin (CT). Ab titers were measured in pooled plasma samples (5 mice/group) collected three weeks after secondary and tertiary immunization. It is evident that M2e(pep)-specific Abs accounted for the majority (79% ± 18%, SD) of the total G40d-specific response (defined in each sample as 100%). M2e(pep-nat)-specific Abs made up a smaller and more variable fraction (10% ± 8%, SD) of the total G40d-specific response. In most experiments, only M2e(pep)- and M2e(pep-nat)-specific Ab titers were determined. Taking 27 distinct vaccination groups into account, M2e(pep-nat)-specific Ab titers ranged from ~1% to essentially 100% of the M2e(pep)-specific Ab titers and accounted on average for 14.5% (geometric mean, GM) of the M2e(pep)-specific response (Fig 2C). The various immunization protocols employed here had no significant effect on the size of the M2e(pep-nat)-specific Ab fraction (Fig 2C).

Taken together, the results indicated that the majority of the M2e-MAP-induced Abs were M2e(pep)-specific, and that a variable fraction of these Abs crossreacted with M2 expressed by HeLa-M2 cells, i.e. displayed M2e(pep-nat)-specificity.

### Roles of adjuvant and immunization route on Ab response and protection

**Figure 3:**
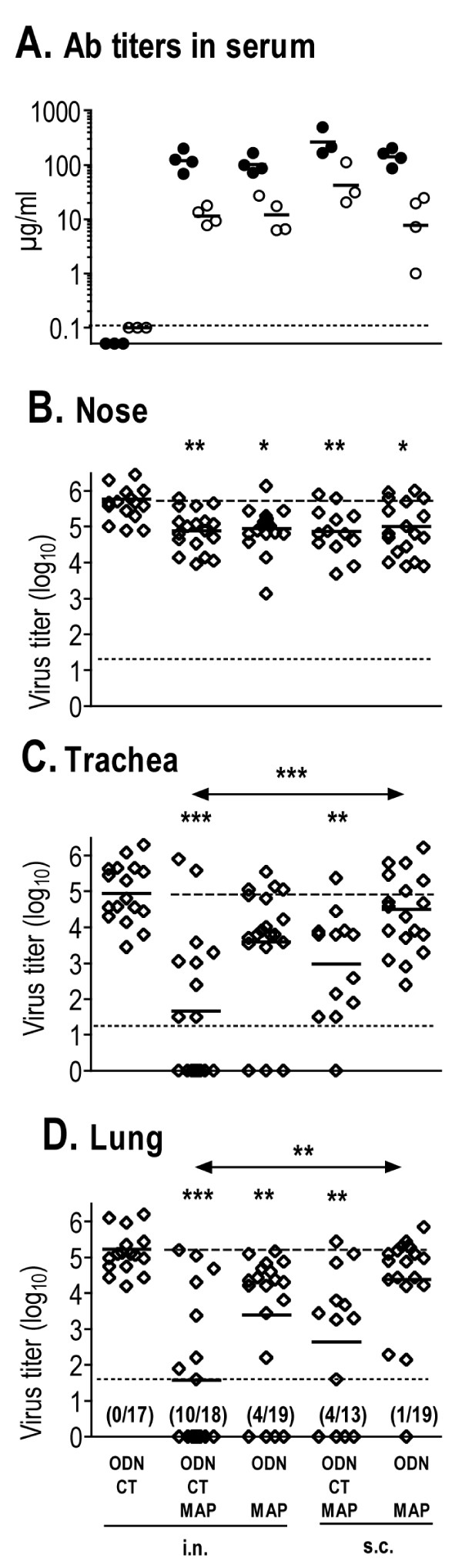
**Ab response and protection after various modes of vaccination**. A. BALB/c mice were vaccinated three times at 4–5 week intervals with 3 μg M2e-MAP (two experiments G39d, two G40d) and the indicated adjuvants (see bottom of figure) by i.n. or s.c. route. Mice were bled 3 weeks after the third immunization. Pooled plasma samples (3–5 mice/pool) were tested by ELISA for M2e(pep)- (dots) and M2e(pep-nat)-specific (circles) Ab titers. Horizontal bars indicate GMTs within each set. Data from four independent vaccination experiments are shown. B, C, D. 7–10 days after the third vaccination, mice were challenged by i.n. inoculation of 5 μl X31 (1000 TCID_50_/mouse). Five days later, nose, trachea and lung were tested for virus titer. Each symbol indicates the virus titer of an individual mouse. Horizontal bars indicate the GMT within each vaccination set. Dashed (top) and stipulated (bottom) horizontal lines indicate the mean virus titer of control mice and threshold of virus detection, respectively. Tissues with undetectable virus were assumed to be virus free. Data were analyzed by non-parametric ANOVA and Dunn's Multiple Comparison post test. M2e-MAP vaccination groups with statistically significant reduction in virus titer compared to the control group are indicated by asterisks right above the group and statistical differences between M2e-MAP vaccination groups by asterisks above two-sided arrows: p < 0.05 (*), p < 0.01 (**), p < 0.001 (***).

In our previous study [17], we had shown that mice vaccinated with M2e-MAP, ODN and CT by the i.n. route exhibited significant resistance to total respiratory tract infection with IAV. Here, we wanted to determine whether route of vaccination and use of CT as adjuvant made a significant contribution to protection. To this end, mice were immunized three times at 4–5 week intervals with M2e-MAP plus ODN with or without CT by i.n. or s.c. (tail base) routes. M2e-specific Ab titers in plasma (pools of 4–5 mice per group) collected three weeks after the third immunization were determined and mice challenged 7–10 days later by nasal infection with X31. Results from four independent repeat vaccination and challenge experiments are compiled in Fig 3.

**Figure 4:**
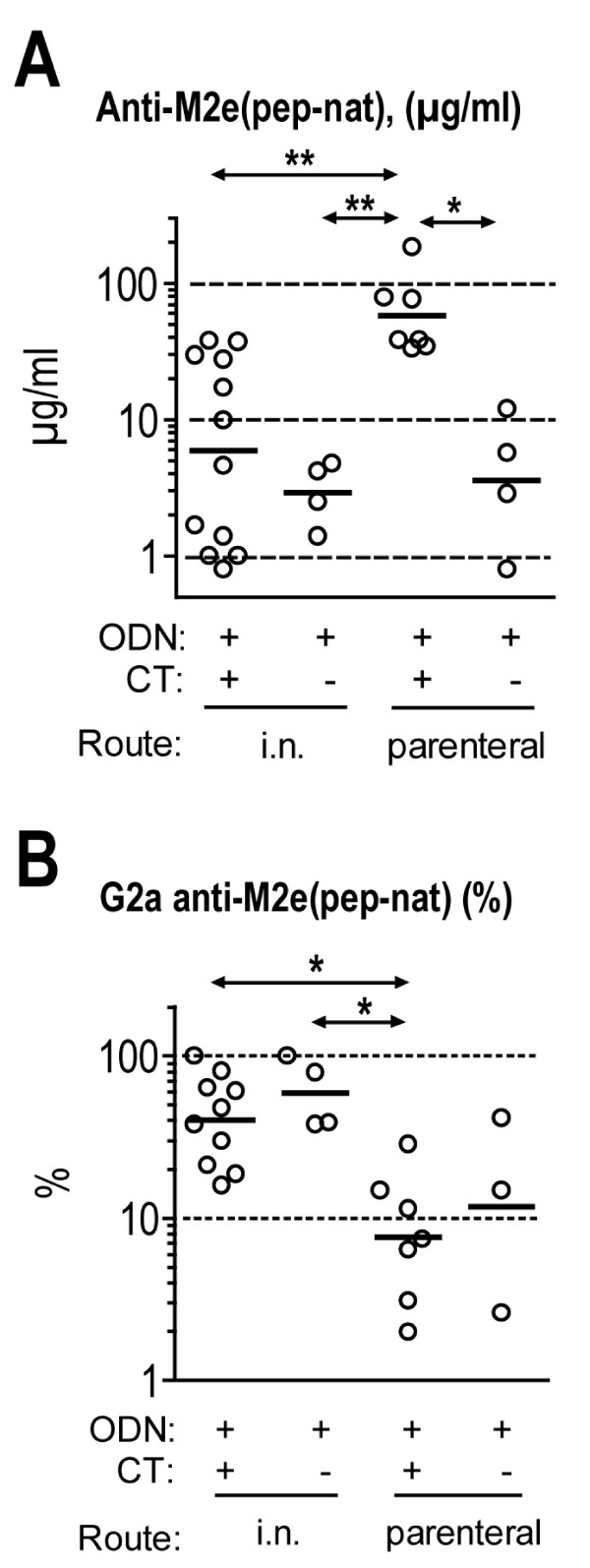
**Effect of immunization protocol on size and G2a content of the M2e(pep-nat)-specific Ab response**. A. M2e(pep-nat)-specific Ab titers in pooled plasma samples collected three weeks after second immunization from mice vaccinated with M2e-MAP according to the protocol indicated below the x axis. Each dot shows the titer of pooled plasma from 3–5 mice. Horizontal bars indicate the GMTs of groups within a given vaccination protocol. Data were analyzed by ANOVA and Tukey's Multiple Comparison post test. Statistically significant differences between group are indicated by asterisks above two-sided arrows: p < 0.05 (*), p < 0.01 (**). B. Pooled plasma from 4–5 mice/group collected three weeks after second and third immunization were tested for concentration of Cκ- (total) and γ 2a-expressing M2e(pep-nat)-specific Ab titers and the latter were expressed as percentage of the former. In groups that were immunized three times, the mean percentage of G2a after 2^nd ^and 3^rd ^immunization is shown. Groups with low M2e(pep-nat)-specific Ab titers that did not permit detection of G2a at ≤5% were excluded from the analysis. Horizontal bars show GMTs within distinct immunization protocols. Data were analyzed by ANOVA and Tukey's Multiple Comparison post test and marked as in A.

As shown in Fig 3A, M2e(pep)- and M2e(pep-nat)-specific Ab titers were slightly higher in mice vaccinated with ODN and CT by the s.c. route than in the other vaccination groups. Although this difference was not significant (by ANOVA) in the four experiments shown in Fig 3A, it was significant when Ab titers after the second immunization were analyzed and additional vaccination experiments taken into account (Fig 4A). Thus, in the presence of ODN, CT strongly enhanced the Ab response upon parenteral though not i.n. vaccination.

The strength of protection was assessed by i.n. inoculation of mice with 5 μl (1000 TCID_50_) of X31 virus. This challenge induces an infection that is initially confined to the nasal epithelium and from there spreads in non-immune mice within a few days into the lower respiratory tract. Five days after challenge, mice were euthanized and virus titers determined in nose, trachea (together with extrapulmonary bronchi) and lung. As shown in Fig 3B–D, the infection had spread by this time in all control mice (immunized with adjuvant only) into trachea and lung. Compared to the control group, all M2e-MAP vaccination groups showed significant restriction of similar strength against virus growth in the nose (Fig 3B). The groups differed, however, with regards to resistance against descending infection. The least resistance was seen in mice vaccinated with M2e-MAP and ODN by the s.c. route and in fact did not differ significantly from the control group. The strongest and most significant resistance was seen in mice vaccinated with ODN and CT by the i.n. route. The other two vaccination groups (i.n. with ODN but without CT and s.c. with ODN and CT) displayed intermediate and similar levels of protection.

Taken together, the results indicated that CT significantly enhanced the systemic Ab response when administered together with ODN by a parenteral route and strengthened protection both upon parenteral and i.n. vaccination. Furthermore, independent of the adjuvants used, the i.n. route of vaccination engendered stronger protection than parenteral vaccination. However, the relationship between strength of protection and M2e-specific serum Ab titer was not clear. For instance, mice vaccinated with M2e-MAP and ODN by s.c. route displayed significantly weaker resistance against descending infection than mice immunized with M2e-MAP, ODN and CT by i.n. route, in spite of similar serum Ab titers. This was unexpected in view of previous findings showing that protection could be transferred with serum from M2e-immune to naive mice [4-6,8,9].

### Relation between M2e-specific serum Ab titers and protection

The absence of a clear relation between serum Ab titer and strength of protection suggested that the concentration of Cκ-positive M2e-specific Abs in serum (biotinylated anti-Cκ was used for measurement of Ab titers) was not the sole determinant of protection. Although λ light chains are expressed only by ~5% of Abs in the BALB/c mouse, they may be expressed at higher frequency in responses of some specificities. We therefore tested selected serum samples for λ-positive M2e-specific Abs but found no evidence for the substantial use of λ light chains in the M2e-specific Ab response (data not shown). Thus, differences in the fine specificity, avidity or heavy chain isotype of M2e-specific serum Abs or of immune phenomena that are mostly confined to the respiratory tract and poorly reflected in serum could make significant contributions to protection. To further explore these possibilities, we analyzed the relation between M2e-specific serum Ab titers and strength of protection in the above and additional groups of mice that had been vaccinated with M2e-MAP, challenged by localized nasal infection with the same dose of X31 virus and analyzed for virus titer five days later. To detect potential contributions of respiratory tract-associated immune phenomena, which may be induced preferentially by i.n. immunization, groups vaccinated by i.n. and parenteral routes were analyzed separately. The reduction in virus titer (on log_10 _basis) in M2e-MAP immunized groups compared to the control group (adjuvant only) of the given immunization experiment was used as measure of strength of protection. Tissues with undetectable virus (threshold of 10 EID_50 _for nose and trachea and 10^1.3 ^for lung) were assumed to be virus-free.

As shown in Table 1, Cκ-positive M2e(pep)-specific serum Ab titers showed no significant correlation with strength of protection, both after i.n. and parenteral immunization. By contrast, highly significant correlations were seen between M2e(pep-nat)-specific Ab titers and protection after parenteral though not i.n. immunization. These findings indicated, firstly, that only Abs capable of reacting with native cell-expressed M2e played a role in protection. Since M2e(pep-nat)-specific Abs are a subpopulation of the M2e(pep) specific response, the absence of correlation between M2e(pep)-specific Ab titers and protection is apparently a consequence of the substantial variation between groups in the proportion of M2e(pep-nat)-specific Abs within the total M2e(pep)-specific response (Fig 2C). Second, the absence of correlation between M2e(pep-nat)-specific serum Ab titers and protection in mice immunized by the i.n. route indicated that M2e(pep-nat)-specific serum Abs were not the sole effectors of protection; conceivably, M2e-specific Abs produced in airway tissues, whose titers are inadequately reflected in serum, or M2e-specific T cells may contribute to protection.

**Table 1:** Correlation between M2e-specific serum Ab titer and reduction of virus titer in various sites of the respiratory tract after parenteral and i.n. immunization.

Specificity/Isotype of anti-M2e Abs	Spearman correlation coefficient r (p)					
	
	parenteral vaccination			i.n. vaccination		
	
	Nose	Trachea	Lung	Nose	Trachea	Lung
M2e(pep)	0.07	-0.03	0.18	0.2	0.24	0.02
M2e(pep-nat)	0.96(***)	0.82 (**)	0.8 (**)	-0.38	-0.27	-0.31
M2e(pep-nat) G2a	0.56	0.51	0.75 (*)	-0.43	-0.30	-0.35

Mean (4–5 mice/group) M2e-specific serum Ab titers 7–10 days before challenge with X31 virus (5 μl, 10^3 ^TCID_50_) were analyzed for correlation with mean reduction (compared to control group) of virus titers in nose, trachea and lung. Included in the analysis are 9 groups of mice immunized with M2e-MAP by a parenteral route (s.c., i.m., i.p.) and 14 groups immunized by the i.n. route. Statistical significance of correlation (Spearman r, non parametric) is indicated by asterisk: (*): p < 0.05, (**): p = 0.01, (***): p = 0.002.

**Figure 5:**
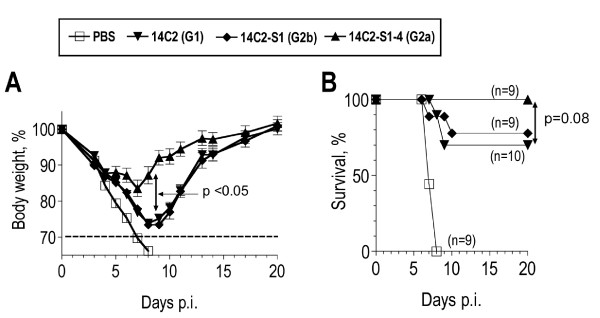
**Role of heavy chain isotype in protection**. Naive BALB/c mice were injected i.p. with 10 μg mAb 14C2 of G1 (triangles pointing down), G2b (diamonds) or G2a (triangles pointing up) isotype. The control group (open squares) received PBS i.p. One day later, mice were exposed to a total respiratory tract challenge with PR8 (4 LD_50 _in 50 μl) and monitored for weight loss. Pooled data from two independent experiments are shown, each performed with 4–5 mice/group. A. Symbols show mean % body weight and SEM (relative to day 0) of 9–10 mice/group. Differences between treatment groups were tested for statistical significance at individual days. Mice treated with G2a showed significantly (p < 0,05, ANOVA) less weight loss than those treated with G1 or G2b at days 6 to 13 p.i. B. Survival. Death was defined as >30% weight loss, at which stage mice were euthanized. Differences between survival curves were tested for statistical significance by log rank test.

Abs of G2a isotype have often been found to display higher activity in vivo than Abs of other IgG isotypes. This has been attributed to the ability IgG2a to interact with all three activating IgG Fc receptors, FcγRI, FcγRIII and most notably FcγRIV, for which G2a is the preferred isotype[19,20]. In agreement with this, naive mice, passively protected with the G2a isotype switch variant of mAb 14C2, showed significantly less weight loss (p < 0.05) and less mortality (p = 0.08) than mice passively protected with the same dose of mAb 14C2 of G1 or G2b isotype (Fig 5). Therefore, we determined also titers of M2e(pep-nat)-specific G2a in sera, hoping Abs of this isotype may show an improved correlation with protection. However, the contrary was the case, possibly because positive effects on correlation due to the increased protective activity of G2a were outweighed by negative effects on correlation due to the variability in the proportion of G2a within the total M2e(pep-nat) response (Fig 4B). It is possible also that the G2a isotype provides a smaller advantage over other isotypes in inhibition of a descending infection by X31 virus – the endpoint used for the data in table 1 – than in reduction of morbidity and mortality after total respiratory tract challenge with PR8 – the endpoint used in the comparison of the isotype switch variants (Fig 5). An interesting observation resulting from this analysis was that i.n. vaccination engendered an Ab response with a significantly larger proportion of G2a (GM: 45%) than parenteral immunization (GM: 8%), independent of the adjuvants used (Fig 4B).

**Figure 6:**
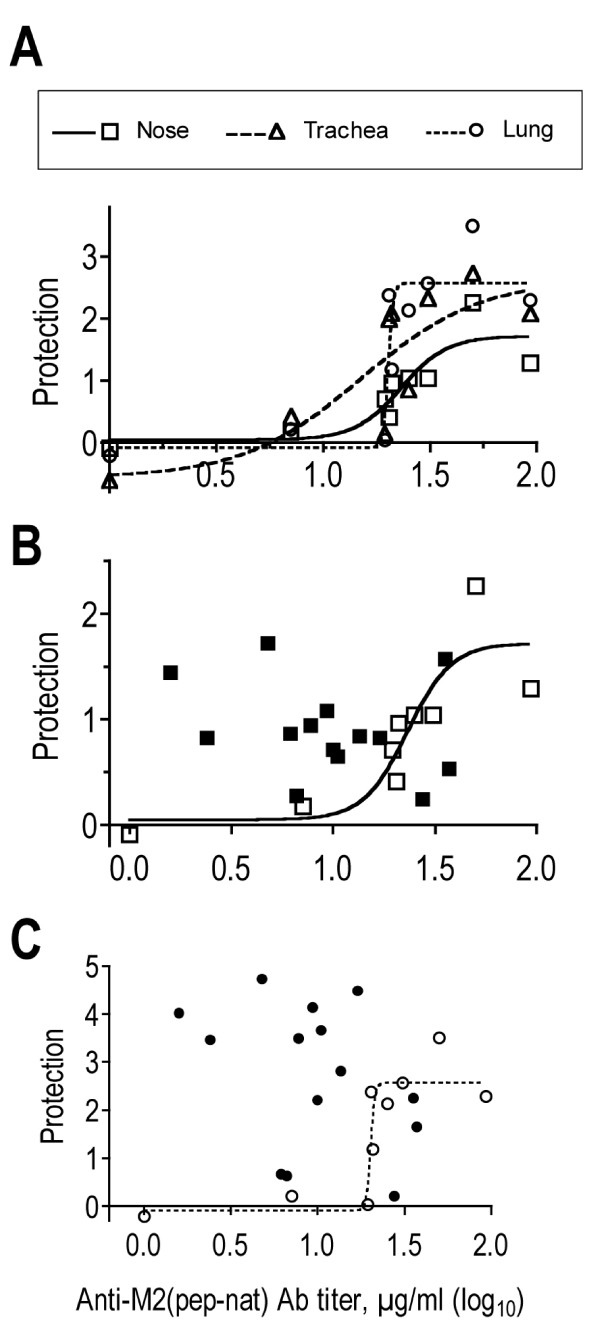
**Relation between M2e(pep-nat)-specific Ab titer and protection against virus challenge**. Cκ-positive M2e(pep-nat)-specific Ab titers were determined in pooled plasma (3–5 mice/group) collected 7–10 days before challenge of mice by localized nasal infection (5 μl X31, 1000 TCID_50_). Five days after challenge, virus titers were determined in nose, trachea and lung of individual mice and the group average was determined. The average reduction in virus titer on log_10 _basis compared to the control group (immunized with adjuvant alone) was taken as measure of strength of protection (y axis). A. Protection in nose (squares), trachea (triangles) and lung (circles) from nine groups of mice immunized by parenteral route is plotted against the M2e(pep-nat)-specific serum Ab titer (x axis). Non-linear regression analysis yielded sigmoidal regression curves with R^2 ^of 0.79 for nose (stipulated) and lung (continuous) and of 0.65 for trachea (dashed). B. Serum Ab titers and protection in nose observed in mice immunized by the i.n. route (filled squares) are plotted together with the regression line and corresponding data points (open squares) from mice immunized by parenteral route (as in A). C. Serum Ab titers and protection in lung of mice immunized by i.n. route (filled circles) are plotted together with the regression line and corresponding data points (open circles) from mice after parenteral immunization (as in A).

In view of the significant correlation between total M2e(pep-nat)-specific serum Ab titer and protection after parenteral immunization, we subjected the data to linear and non-linear regression analysis. Linear regression analysis showed a poor fit between Ab titer and protection, with R^2 ^values of 0.45, 0.36 and 0.37 for protection in nose, trachea and lung, respectively, though elimination of one outlier group with the highest serum Ab titer yielded linear regressions with R^2 ^and (p) values of 0.94 (<0.0001), 0.66 (0.014) and 0.78 (0.0039) for protection in nose, trachea and lung, respectively. However, without exclusion of any data, the relations between Ab titers and protection could be described by sigmoidal curves that exhibited R^2 ^values of 0.79 for nose and lung and 0.65 for trachea (Fig 6A). They indicated that M2e-specific protection after parenteral immunization exhibited an upper boundary and half-maximm protection was achieved in each site of the respiratory tract at the serum Ab concentration of ~20 μg/ml. By contrast, in i.n. vaccinated mice, there was no obvious relation between serum Ab titer and protection. Indeed, significant protection was seen in many mice with serum Ab titers that were completely non-protective in parenterally vaccinated mice. This is demonstrated in Fig. 6B and 6C, which display the data from individual i.n. (filled symbols) and parenterally vaccinated (open symbols and deduced sigmoidal curve) groups for protection in the nose and lung, respectively. Apparently, vaccination by the i.n. route was capable of inducing potent protective activities other than those mediated by M2e-specific serum Abs.

### Ab response and protection after i.n. administration of M2e-MAP together with infectious virus

Recovery from respiratory tract infection has been shown to result in optimal protection [21]. This is generally attributed to the combined effects of strong local and systemic T and B cell responses against several viral proteins. Since infection induces only a poor M2e-specific Ab response [13], we wondered whether infection-induced protection could be further strengthened by concomitant immunization with M2e-MAP. This was tested by i.n. administration of a sublethal dose of PR8, either alone or together with 3 μg M2e-MAP. Both groups of mice developed a primary infection from which they recovered. Four weeks later, the mice were inoculated i.n. with PR8-Seq14 (200 TCID_50_), again with or without M2e-MAP. Other groups of mice were inoculated twice by the i.n. route with A) ODN and CT (negative control), B) M2e-MAP (3 μg/dose) in PBS without adjuvant, C) M2e-MAP with ODN and CT (positive control) or D) 5 μg purified uv-inactivated PR8 virus. We decided on this dosage of inactivated virus, which contains ~10^6 ^times the amount of virus present in 200 TCID_50_, to compensate for the lack of replication in vivo. Plasma samples were collected three weeks after the boost, pooled within each group, and tested by ELISA against M2e peptide and HeLa-M2.

**Figure 7:**
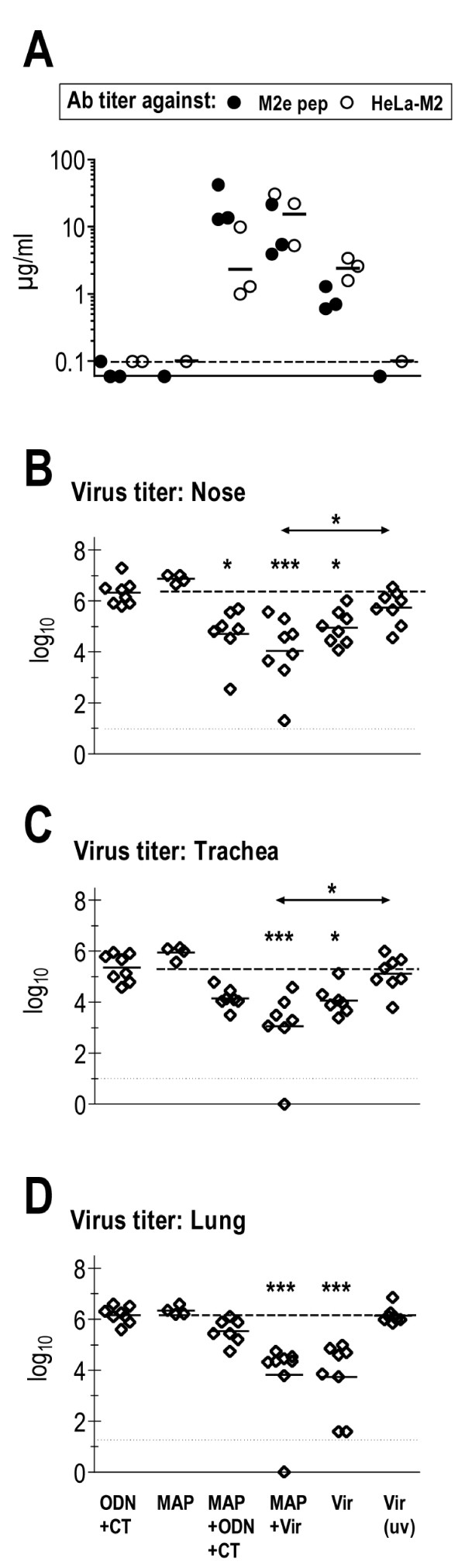
**Immunization with the combination of infectious virus and M2e-MAP**. BALB/c mice were immunized twice (4 week interval) by the i.n. route with the components listed at the bottom of the figure. Dosage/injection (50 μl): M2e-MAP G39d (3 μg), ODN (3 μg), CT (0.5 μg), Vir (150–200 TCID_50 _of PR8 for primary and of Seq14 for secondary immunization), Vir(uv) (5 μg of purified uv-inactivated PR8, <1 TCID_50_). Plasma was collected three weeks after second immunization and pooled within groups. A. Ab titer measured by ELISA against M2e peptide (closed circles) and HeLa-M2 (open circles) in pooled plasma samples of groups of 3–4 mice from three independent vaccination experiments. Bars indicate the GMTs. The stipulated horizontal line indicates the threshold of detection of Ab titers against HeLa-M2. B, C, D. Four weeks after the second immunization, mice from two vaccination experiments were challenged by i.n. inoculation of 50 μl X31, which initiates an infection throughout the entire respiratory tract. Virus titers in nose, trachea and lung were determined three days later. Each symbol indicates the total virus titer (TCID_50_) from an individual mouse in the nose (B), trachea (C) and lung (D). Bars indicate GMTs. The data were analyzed by non-parametric ANOVA and Dunn's Multiple Comparison Test. Statistical significance between experimental and control groups and between experimental groups is indicated by asterisks above each column and above two-sided arrows, respectively: p < 0.05 (*); p < 0.01 (**).

Fig 7A shows Ab titers from three independent vaccination experiments. No M2e-specific Abs were detected in sera of mice immunized with M2e-MAP without adjuvant or with inactivated virus. The former shows that M2e-MAP is not immunogenic in the absence of adjuvant and the latter that M2 – a minor viral structural protein that makes up only ~0.2% of the total protein mass of virus particles [22] – is not immunogenic in the context of a large dose of mature virus particles. Note, however, that mice immunized twice with inactivated virus made a strong HA-specific Ab response (data not shown). M2e-specific Ab responses were seen in all other groups. However, they differed in titer and fine specificity in that mice immunized with M2e-MAP plus adjuvant displayed higher Ab titers against M2e peptide than HeLa-M2 while, as shown previously [13], the reverse was the case for mice immunized by infection. Importantly, the highest Ab titers against HeLa-M2 were seen in mice immunized concomitantly with infectious virus and M2e-MAP, and the majority of these Abs appeared to display M2e(pep-nat)-specificity.

Seven to fourteen days after the second immunization, mice from two vaccination experiments were challenged by i.n. instillation of 50 μl of X31. This mode of challenge initiates an infection throughout the respiratory tract (nose, trachea, pulmonary airways) and was chosen in preference of the localized nasal infection because the latter did not descend within five days into the lower respiratory tract in infection-immunized mice (our unpublished observation) and therefore was unsuitable for revealing differences between the groups immunized by infection with/without M2e-MAP. Three days after total respiratory tract infection, mice were euthanized and virus titers in nose, trachea and lung determined. Compared to control mice (adjuvant alone), significant reductions in virus titers were seen in mice immunized with M2e-MAP plus adjuvant, M2e-MAP plus infectious virus or infectious virus alone, but the two infection-immunized groups showed stronger protection in the lung than the M2e-MAP/adjuvant-immunized group. Comparison between the infection-immunized groups indicated a slight increase in resistance against virus replication in the nose and trachea in mice that had been co-immunized with M2e-MAP, although the difference did not reach statistical significance with the few mice used in these experiments.

Taken together, the results indicated that combined vaccination with M2e-MAP and infectious virus may improve the induction of HeLa-M2-reactive Abs and slightly enhance protection in nose and trachea compared to vaccination with infectious virus or M2e-MAP alone.

## Discussion

### Relation between Ab specificity, titer and protection

We found that the concentration of M2e(pep-nat)-specific Abs in sera of parenterally vaccinated mice correlated with strength of protection (Table 1). This is consistent with previous studies showing that protection can be transferred to naive mice by passive M2e-specific Abs [10,11,17] and antisera [4-6,8,9]. It is consistent also with the generally held view that M2e-specific Abs mediate protection by reaction with M2e expressed in the plasma membrane of infected host cells. By contrast, M2e(pep)-specific Ab titers showed no correlation with protection, in spite of the fact that the M2e(pep-nat)-specific Abs are a fraction of the larger M2e(pep)-specific Ab response (Table 1). This lack of correlation between M2e(pep) Ab titer and protection appears to be a consequence of the large variability of the M2e(pep-nat)-fraction within the M2e(pep) response (Fig 2C). The reason for this variability is not known. It could be due to a low frequency of M2e(pep)- and M2e(pep-nat)-specific B cells within the naive B cell repertoire, which may result, for stochastic reasons, in large differences in the composition of this response between individuals and even pooled sera from 3–5 animals, as tested here. A low precursor B cell frequency is consistent with our previous finding that seven M2e(pep-nat)-specific hybridomas isolated from three mice all expressed a highly restricted heavy chain variable region (formed by recombination of the same V_H_, D and J gene segments) in association with only two distinct V_L _genes [23]. The M2e(pep)-specific response has not been analyzed at the clonal level but may be equally restricted. Because of this variability, the protective M2e(pep-nat)-specific Ab titers cannot be extrapolated from M2e(pep)-specific Ab titers and must be measured specifically.

Given the importance of M2e(pep-nat)-specific Abs in protection, the selective promotion of this Ab population by a M2e vaccine would be advantageous. This may be achieved by development of a more effective vaccine construct and/or vaccine administration. Of note in the latter context is the present finding that concomitant administration of M2e-MAP and a sublethal dose of infectious virus by the i.n. route not only enhanced the M2e(pep-nat)-specific serum Ab titer compared to vaccination with infectious virus or M2e-MAP (plus adjuvants) alone, but affected also the specificity of the response in that essentially all M2e-specific Abs generated in these co-immunized mice displayed M2e(pep-nat)-specificity (Fig 7A and data not shown). The advantage of co-administration of infectious virus and M2e-MAP with regard to strength of protection against heterosubtypic IAV challenge (as used in the present study) merit further investigation, particularly since this protocol may be adaptable to humans in the form of i.n. vaccination with a combination of live attenuated IAV and a M2e-vaccine.

The relation between M2e(pep-nat)-specific Ab titers in sera of parenterally vaccinated mice and strength of protection followed sigmoidal curves (Fig 6A), which suggested that M2e(pep-nat)-specific serum Abs were equally protective in nose, trachea and lung (EC_50_~20 μg/ml). This was unexpected in view of previous studies showing that systemically administered passive anti-viral Abs of IgG isotypes were significantly less protective in upper than lower airways [24-26]. The reason for this appears to be the lower rate of transudation of serum IgG through the pseudostratified columnar epithelium of upper airways than the thinner epithelium of respiratory airways and alveoli [27]. To confirm that this differential effectiveness applies also to M2e(pep-nat)-specific Abs, we injected fifteen naive BALB/c mice with three different purified mAbs (5 mice/Ab) to achieve a passive serum Ab concentration of ~20 μg/ml and then challenged the mice by i.n. inoculation of 5 μl X31. Determination of virus titers in lung, trachea and nose five days later confirmed the decreasing protective activity of serum Ab from lower to upper airways, in that mAb-treated mice exhibited, on average, a 100 fold reduction in virus titer in the lung, 30 fold in the trachea and no reduction at all in the nose compared to control mice treated with PBS (data not shown). Accordingly, M2e(pep-nat)-specific serum Ab titers in mice that had been immunized by a parenteral route appeared to account reasonably well for the protection in lung and trachea but not in the nose.

One possible explanation for this difference in protection between actively and passively immunized mice was that active immunization induced substantial levels of M2e(pep-nat)-specific IgA. When dimerized with J chain, IgA is actively transported by the polymeric Ig receptor (pIgR) system through the columnar epithelium of conducting airways and is therefore more abundant than IgG in secretions of upper than lower airways [27,28]. Accordingly, secretory IgA with virus-neutralizing activity has been shown to be responsible for much of the protection against IAV replication in the nasal cavity of mice, while IgG is more important for protection of respiratory airways [29-31]. However, we could not detect significant levels of M2e(pep-nat)-specific IgA in sera of parenterally vaccinated mice (data not shown), making this explanation untenable. Another possibility, which is discussed in more detail below, is that parenteral vaccination with M2e-MAP induced significant airway-associated immunity. Although induction of strong local airway-associated immunity is generally thought to require administration of antigen into the airways [31-33], there is evidence indicating that parenteral immunization with CT may result in the migration of dendritic cells to mucosa-associated lymphoid tissues and thereby promote some level of mucosa-associated immunity [34,35]. In this study, CT significantly enhanced the systemic Ab response upon parenteral vaccination but we do not know whether it also resulted in the induction of nasal mucosa-associated immunity that may have restricted virus replication in nasal tissue, independent of serum Ab titer. Finally and probably most likely, immunization with M2e-MAP may have induced not only M2e-specific Abs but also T cells that contributed to protection. This possibility is supported by previous studies showing that vaccination of BALB/c mice with M2e-MAP [17] or M2-DNA and M2-recombinant adenovirus [9] induced M2e-specific T cell responses, most likely of CD4 phenotype, and that virus-specific CD4 memory T cells could significantly restrict virus replication in the nose but not the lung [36]. Accordingly, M2e-specific CD4 T cells may have inhibited virus replication in the nose and M2e-specific serum Abs in the lung. This proposition does not conflict with the conclusion of Jegerlehner et al. [6] that M2e-specific T cells played no role in protection of mice against a lethal total respiratory virus challenge, as the lethality of the infection is determined by the level of virus replication in the lung but not the nose. The contrasting finding by Tompkins et al. [9] that T cells contributed to protection against lethal IAV challenge in mice immunized by M2-DNA and M2-recombinant adenovirus may be explained by induction of M2-specific CD8 T cells in these mice. It is well established that virus-specific memory CD8 T cells can contribute to resistance against a lethal IAV challenge.

### Route of vaccination and strength of protection

I.n. vaccination resulted in stronger protection against descending infection than parenteral vaccination (Fig 3C,D). Most remarkably, however, the strength of protection in i.n. vaccinated mice showed no correlation with M2e(pep-nat)-specific serum Ab titers (Table 1). Indeed, several groups of i.n. vaccinated mice with serum Ab titers that were completely non-protective in parenterally vaccinated mice showed nevertheless strong protection (Fig 6B,C). Several explanations can be considered.

First, i.n. administration of adjuvant alone has been shown to result in a temporary increase in resistance against virus replication in the respiratory tract [37-40]. However, such a non-specific enhancement of resistance is unlikely to have affected the results of this study, since M2e-MAP-vaccinated mice were always compared to control mice that had been vaccinated by the i.n. route with adjuvant alone, thus canceling out adjuvant-induced non-specific effects.

Second, i.n. vaccination may have induced local, airway-associated immunity that was not adequately reflected by serum Ab titers. To affect virus replication, Abs must be present in airway secretions. Abs in this location may have two distinct provenances [41]: 1) They may be serum Abs that transudated into extravascular spaces of airway tissues and, in the case of IgG, transudated further into the airway lumen or, in the case of IgA and IgM, became transported through the epithelial cell layer by pIgR. 2) They may have been secreted by B cells located in the lamina propria of airways. As such locally produced Abs, particularly J-chain associated IgA and IgM, can be expected to be delivered more effectively into the airway lumen than into the intravascular compartment, serum Ab titers do not provide a reliable measure of the locally produced fraction of Abs. The importance of nasal administration of vaccine for promotion of local immunity has been documented both in animal models [31,42-45] and humans [46-49]. Once induced, antigen-specific B and T cells may persist in airway tissues for an extended period of time and provide the host with long lasting enhanced protection [50-54]. Accordingly, local M2e(pep-nat)-specific B and possibly also T cells may have provided strong protection in some i.n. vaccinated mice in the absence of protective serum Ab titers (Fig 6B,C).

Third, i.n. vaccination may have induced a qualitatively different and more protective immune response than parenteral vaccination. It is well established, for instance, that i.n. vaccination typically promotes a stronger IgA response than parenteral vaccination. The fact that we could not detect significant M2e(pep-nat)-specific IgA in pooled sera of i.n. vaccinated mice (data not shown) does not exclude the possibility that M2e(pep-nat)-specific IgA was produced locally and efficiently transported into airway secretions. In contrast to IgG, locally produced IgA may interact intracellularly with M2e during its pIgR-mediated transport through infected epithelial cells and thereby restrict virus replication [55]. The substantial efficacy of this mechanism in vivo has been demonstrated by passive IgA mAb-mediated clearance of Rotavirus from intestinal epithelium of mice with severe combined immunodeficiency [56]. After its release into airway secretions, secretory M2e(pep-nat)-specific IgA may have lesser protective power than IgG, both in terms of activation of FcR-expressing effector cells and complement. Nevertheless, cell-bound secretory IgA, while incapable of activating effector cells through one of the widely expressed activating FcγRs, may still be able to activate effector cells through interaction with the recently identified FcαμR in mice [57] or CD86 in humans. In addition, while incapable of activating complement through the classic pathway, IgA may still activate it through the alternative [58] and lectin [59] pathways if complement activation were involved in M2e-Ab-mediated protection. Another potentially important qualitative change observed here after i.n. administration of vaccine was the significant increase in the proportion of M2e(pep-nat)-specific Abs of G2a isotype (Fig 4B). Firstly, IgG2a was the most protective IgG isotype in passive transfer experiments (Fig 5). In addition, if T cells contributed to protection, the prevalence of IgG2a may indicate a general bias of the response towards type 1, which is typically associated with optimal T cell-mediated protection in viral and bacterial infections. Additional studies are needed to sort out the relative importance of local immunity and quality of the response in the improved protection after i.n. vaccination.

The enhanced protection seen here after i.n. vaccination must be viewed in the context of the challenge used here. It consisted of an infection that was initially confined to the nasal epithelium and allowed to descend from there into the lower respiratory tract over the course of five days. In this scenario, strong immunity in the upper respiratory tract would be expected to have a substantial impact on the progress of the infection. By contrast, the more frequently used challenge with an inoculum of 30–50 μl in anesthetized mice initiates an infection in both upper and lower respiratory tact, and virus titer in lung or survival would hardly if at all be affected by immunity in the upper respiratory tract. We believe this nasal challenge provides a relevant model for the IAV infection in humans.

## Conclusion

M2e-MAP is an effective immunogen as roughly 80% of the total M2e-MAP-specific Ab response displayed M2e(pep) specificity. A variable fraction (on average 15%) of these M2e(pep)-specific Abs cross-reacted with presumably native tetrameric M2e expressed by M2-transfected HeLa cells, and the concentration of these M2e(pep-nat)-specific Abs in sera of parenterally immunized mice showed a good correlation with protection against virus challenge. However, M2e(pep-nat)-specific serum Abs did not appear to fully account for protection, particularly in the nose, of M2e-MAP-vaccinated mice, suggesting the contribution of additional protective activities, possibly M2e-specific T cells and/or local airway-associated Ab responses. The latter was supported also by the observation that immunization by the i.n. route resulted in stronger protection than immunization by a parenteral route and that the strength of protection in i.n. vaccinated mice showed no correlation with M2e(pep-nat)-specific serum Ab titers. Concomitant i.n. administration of M2e-MAP with infectious virus enhanced the M2e(pep-nat)-specific Ab response and protection compared to i.n. vaccination with M2e-MAP plus adjuvant or infectious virus alone. Concomitant i.n. administration of M2e-MAP and attenuated cold-adapted live virus may be applicable to human vaccination and merits further investigation.

## Methods

### Mice

Female BALB/c mice (5–6 week old) were purchased from Harlan [60] and maintained in the Institute's Animal Facility in microisolator cages under specific pathogen-free conditions. Mice were rested for ≥2 weeks before use in experiments. All procedures performed on animals were approved by the Institutional Animal Care and Use Committee.

### Media, solutions and reagents

ISC-CM is Iscove's Dulbecco medium (Invitrogen) supplemented with 0.05 mM 2-mercaptoethanol, 0.005 mg/ml transferrin (Sigma), 2 mM L-glutamine (Mediatech Inc) and 0.05 mg/ml gentamicin (Mediatech Inc). ISC-CM was further supplemented, as indicated, with fetal bovine serum (FBS, Gemini Bio-products) or bovine serum albumin (BSA, Sigma). PBSN is phosphate buffered saline (pH7.2) supplemented with 3 mM NaN3. Immunostimulatory phosphorothionated oligodeoxynucleotide (ODN) 1826 [61] and cholera toxin (CT) were purchased from Sigma. Multiple antigenic peptides (MAPs) were synthesized in house [62] and have been described previously [17]. The MAPs used here are: G39d (dimer of disulfide-linked MAPs, each containing two M2e(2–25)-peptides and two helper T cell peptides, one (S1) presented by A^d ^and the other (S2) by E^d ^[63], linked to the Lys of a Cys-(Gly-Lys)_4_-Ala scaffold peptide); G40d (as G39d but with only one helper T cell peptide linked to a Cys-(Gly-Lys)_3_-Ala scaffold peptide); Cys-M2e (two M2e peptides linked to Cys-(Gly-Lys)_3_-Ala) and Cys-bb (Cys-(Gly-Lys)_3_-Ala. G39d was used for all but two immunizations. Fig 1 shows the composition and sequence of the MAPs.

### Monoclonal Abs

The M2e-specific hybridoma 14C2 (IgG1) was originally obtained from Zebedee and Lamb [22]. The 14C2 switch variant of G2b isotype was selected by staining 20 million parental (IgG1) hybridoma cells with rat-anti-mouse-G2b mAb (R1.3-20), sorting by flow cytometry for the 1% most intensively stained cells, culturing the sorted cells by limiting dilution and testing growing cultures for secretion of IgG1 and IgG2b. A G2a switch variant (14C2-S1-4) was similarly selected from the G2b switch variant (14C2-S1) by using the rat-anti-mouse-G2a mAb (G2a-3-6.8) for staining of 14C2-S1 cells. MAbs were purified from protein-free hybridoma medium PFHM-II (Gibco), in which hybridoma cells, initially grown up in ISC-CM 5%FBS, had been cultured to exhaustion. Purified 14C2-S1-4 mAb was used as standard for determination of M2e-specific Ab concentration by ELISA.

### Viruses

PR/8/34(H1N1)-Mt.Sinai (PR8) is a highly pathogenic mouse-adapted IAV. 500 TCID_50 _(50% tissue culture infectious dose) correspond to ~1 LD_50 _(50% lethal dose) when administered in 50 μl into the respiratory tract of anesthetized mice. PR8-Seq14 is an escape mutant derived from PR8 through fourteen sequential selection steps, each performed in the presence of a distinct PR8-specific mAb that was capable of neutralizing the penultimate escape mutant. PR8-Seq14 differs from PR8 by 15 amino acid substitutions in HA, is not measurably inhibited in hemagglutination inhibition assay by PR8-specific mouse sera and retains the high pathogenicity of PR8 (observations to be published). X31 is a reassortant between PR8 and A/Aichi/68(H3N2). It contains all PR8-derived genes except those encoding H3 and N2 and is of low pathogenicity.

### Immunization, infection and analysis of mice

For i.n. immunization, 50 μl of vaccine preparation (PBS containing 3 μg M2e-MAP, 3 μg ODN 1826 and 0.5 μg CT) was applied to the nares of mice anesthetized by intraperitoneal (i.p.) injection of 0.2 ml ketamine-xylazine (70/7 mg/kg); this results in the aspiration of the vaccine into upper and lower airways. For subcutaneous (s.c.), intramuscular (i.m.) and i.p. immunization, 50 μl of vaccine as above was injected into the tail base, quadriceps or peritoneal cavity, respectively, of non-anesthetized mice. Vaccinations were repeated once or twice, typically in four week intervals. Three weeks after primary, secondary or tertiary vaccination, 0.1–0.2 ml blood was collected by puncture of the retro-orbital plexus, and plasma samples were pooled within each immunization group. Four weeks after the last vaccination, anesthetized mice were challenged by administration of 5 μl PBS (containing 2000 TCID_50 _of X31) to the nares, dispensing ~half of the inoculum per nare. This challenge results in a sublethal infection that is initially confined to the nasal epithelium and then descends in naive mice over the next five days into trachea and lung. Five days after infection, mice were euthanized by exsanguination under ketamine/xylazine anesthesia. Nose, trachea with attached extrapulmonary bronchi and lungs were individually dissected and stored frozen for subsequent determination of infectious virus titer by MDCK assay as described [36]. Tissue homogenates that scored negative in the MDCK assay (sensitivity threshold: 10^1.8 ^TCID_50 _per total nasal and tracheal extract and 10^2.1 ^TCID_50 _per lung extract) were tested by inoculation of 50 μl undiluted tissue extracts into the allantoic cavity of two embryonated chicken eggs (sensitivity threshold: 10 EID_50 _for nose and trachea and 20 EID_50 _for lung). In some experiments, anesthetized mice were challenged by i.n. administration of PR8 in 50 μl PBS; this procedure initiates an infection of upper and lower airways and, depending on virus dose, may be lethal. Infected mice were euthanized three days later for determination of virus titers in airway tissues, or were observed for weight loss.

### Determination of M2e-specific Ab concentration by ELISA

For measurement of Abs specific for cell-expressed (presumably native tetrameric) M2e, we used HeLa cells stably transfected with M2-expressing (HeLa-M2) or empty control plasmid (HeLa-C10) as specific and non-specific (background) immunosorbent, respectively [13]. The specific and non-specific immunosorbents used for measurement of M2e-peptide-specific Ab concentration were Cys-M2e and Cys-bb (see above), respectively [17]. Cys-M2e and Cys-bb were used at equimolar concentration (85 nM in 0.02 M NaCl) to coat wells of polyvinyl plastic plates (25 μl/well, overnight at room temperature, under cover to prevent evaporation). For measurement of total M2e-MAP-specific Ab concentration, we used wells coated as above with M2e-MAP as specific and uncoated wells (only blocked with BSA) as non-specific immunosorbent. Immunosorbent-bound Abs were detected with biotinylated mAb 187 (ATCC HB58, rat-anti-mouse κ chain) or G2a-3-6.8 (rat-anti-mouse-G2a) for measurement of Cκ- or G2a-expressing Abs, respectively. The difference in OD (ΔOD) between specific and non-specific immunosorbent was used for quantification of Ab concentration by comparison to ODs observed with known concentrations of purified M2e-specific mAb 14C2-S1-4 (G2a/Cκ) bound to the same immunosorbents. ELISA data were collected with the e-max ELISA reader and analyzed with Softmax Pro software (both Molecular Devices, Sunnyvale, CA).

### Statistical analyses

Prism 4 software [64] was used for plotting and statistical analysis of data as indicated in figure legends.

## Competing interests

The author(s) declare that they have no competing interests.

## Authors' contributions

KM collected blood samples from vaccinated mice, and performed ELISAs and virus titrations. DZ performed the passive protection studies. GK and LO synthesized the M2e-MAPs. WG designed the studies, immunized mice, analyzed data and wrote the manuscript. All authors have read and approved the manuscript.
